# Malignancy diseases and kidneys: A nephrologist prospect and updated review

**DOI:** 10.1097/MD.0000000000033505

**Published:** 2023-04-14

**Authors:** Elmukhtar Habas, Raza Akbar, Kalifa Farfar, Nada Arrayes, Aml Habas, Amnna Rayani, Gamal Alfitori, Eshrak Habas, Yaqeen Magassabi, Hafidh Ghazouani, Aisha Aladab, Abdel-Naser Elzouki

**Affiliations:** a Facharzt Internal Medicine, Facharzt Nephrology, Medical Department, Hamad General Hospital, Doha, Qatar; b Medical Department, Hamad General Hospital, Doha, Qatar; c Facharzt Internal Medicine, Medical Department, Alwakra General Hospital, Alwakra, Qatar; d Medical Education Fellow, Lincoln Medical School, University of Lincoln, Lincoln, UK; e Hematology-Oncology Department, Tripoli Children Hospital, Tripoli, Libya; f Facharzt Pediatric, Facharzt Hemotoncology, Hematology-Oncology Department, Tripoli Children Hospital, Tripoli, Libya; g Medical Department, Tripoli Central Hospital, University of Tripoli, Tripoli, Libya; h al-Arqam Academy, Doha, Qatar; i Quality Department, Senior Epidemiologist, Hamad Medical Corporation, Doha, Qatar; j Hamad General Hospital, Doha, Qatar.

**Keywords:** AKI, cancer-induced AKI, HSCT, hyperphosphatemia, hyperuricemia, TLS, TMA

## Abstract

Acute kidney injury (AKI), chronic renal failure, and tubular abnormalities represent the kidney disease spectrum of malignancy. Prompt diagnosis and treatment may prevent or reverse these complications. The pathogenesis of AKI in cancer is multifactorial. AKI affects outcomes in cancer, oncological therapy withdrawal, increased hospitalization rate, and hospital stay. Renal function derangement can be recovered with early detection and targeted therapy of cancers. Identifying patients at higher risk of renal damage and implementing preventive measures without sacrificing the benefits of oncological therapy improve survival. Multidisciplinary approaches, such as relieving obstruction, hydration, etc., are required to minimize the kidney injury rate. Different keywords, texts, and phrases were used to search Google, EMBASE, PubMed, Scopus, and Google Scholar for related original and review articles that serve the article’s aim well. In this nonsystematic article, we aimed to review the published data on cancer-associated kidney complications, their pathogenesis, management, prevention, and the latest updates. Kidney involvement in cancer occurs due to tumor therapy, direct kidney invasion by tumor, or tumor complications. Early diagnosis and therapy improve the survival rate. Pathogenesis of cancer-related kidney involvement is different and complicated. Clinicians’ awareness of all the potential causes of cancer-related complications is essential, and a kidney biopsy should be conducted to confirm the kidney pathologies. Chronic kidney disease is a known complication in malignancy and therapies. Hence, avoiding nephrotoxic drugs, dose standardization, and early cancer detection are mandatory measures to prevent renal involvement.

## 1. Introduction

Acute kidney injury (AKI) is a frequent complication of cancers that can affect up to 50% of the patients during their course of cancer. It significantly influences the overall prognosis, duration of hospitalization, and consequent healthcare costs. Various factors, including patient type, malignancy type, or therapy, may cause AKI. The patient-related risk factors for AKI are the same as those in the general population. Tumor-related risk factors include compression, blockage, direct kidney infiltration from the tumor, paraprotein precipitation, aggregation, and crystallization in renal tubules. Treatment-related risk factors are the most prevalent in clinical practice. They may manifest as tumor lysis syndrome (TLS), venous-occlusive disease (VOD), or thrombotic microangiopathy (TMA), increasing AKI risk.^[1]^

End-stage renal disease (ESRD) increases malignancy risk in native and transplanted kidneys.^[2,3]^ However, it is unknown whether this increased risk occurs more in the early or late stages of chronic kidney disease (CKD). Depending primarily on the type of cancer and the patient’s age, the effects of CKD may vary.^[4]^ The relationship between cancer and CKD seems to be bidirectional: cancer may induce CKD directly or indirectly via the side effects of medications, and CKD is a known risk for cancer.^[5]^

There is conflicting evidence regarding the relationship between cancer risk and the estimated glomerular filtration rate (eGFR). A Scandinavian study investigated the possible association between renal function and cancer incidence.^[6]^ In moderate to severe CKD, the risk of cutaneous and urogenital cancer was observed to be exceptionally high; however, this can partially be due to bias.^[7]^ Yang et al^[8]^ emphasized that CKD is present in almost 32.4% of newly diagnosed cancer patients. It is especially critical to evaluate the degree of this link for the identification of cancer in patients with CKD, which affects not only their short-term results but also their treatment choices for the underlying disease.^[9]^ Studies have revealed that cancer patients with CKD have an increased mortality risk compared with those without CKD.^[10]^ Another study concluded that CKD patients had a high mortality rate due to liver, urinary tract, and kidney cancers. However, a previous study reported an inverse relationship between eGFR and the death rate from liver cancer. They related this inverse relationship to poor response to therapy or increased cancer incidence.^[10]^ Furthermore, following non-dialyzed-CKD patients for the 20-year, nearly a quarter had developed malignancies. Moreover, elderly males with low eGFR have a higher malignancy risk.^[7]^ Although the reasons for renal failure in cancer are usually multifactorial, the classification of prerenal, renal, and postrenal causes remains clinically relevant.^[11]^

Renal impairment affects approximately 20% of myeloma patients. About 50% of myeloma patients have kidney dysfunction at presentation, and almost 5% may require dialysis, significantly affecting the outcome.^[12]^ Between 1997 and 2001, 58% of ESRD cases caused by cancer were due to myeloma; however, myeloma accounts for only up to 1% of all cancers.^[13]^ A recent Eastern Europe study reported that of the 24 different cancers studied, breast (22%), lung (10%), and colon (10%) cancers were more common in CKD patients compared to the normal population. After the first and the second year of follow-up, the prevalence of CKD (stage 3–5) was (12% and 13%), respectively.^[14]^ Patients with pancreatic cancer (19.6%), renal cancer (50%), and urinary tract cancer (34%) were more likely to have CKD, whereas those with brain tumors (3%) and colon cancer (5.3%) were less likely to have CKD.^[14]^ An eGFR of ≤6 mL/min/1.73 m^2^ was present in 0.7% of the CKD patients who needed renal replacement therapy (RRT).^[14]^ Finally, a multidisciplinary strategy is required to limit the prevalence of cancer-associated kidney complications.

## 2. Aim and method

In this nonsystematic clinical-oriented review, we aim to update the renal complications, pathogenesis, epidemiology, prevention, and related treatments of cancer-associated kidney diseases from a nephrology perspective. PubMed, EMBASE, Google, Scopus, and Google Scholar were searched for original and review articles using different phrases and texts, such as acute renal injury in malignancy, hyperuricemia in cancer, CKD in malignancies, kidney involvement in cancer, RRT in cancer patients, etc.

### 2.1. Ethical approval

The Medical Research Centre in Doha did not have to give ethical approval because it was just a review of articles that had already been published.

## 3. Review

### 3.1. Acute kidney injury

AKI has been defined differently according to the systems used. Nephrological (i.e., Risk, Injury, Failure, Loss of kidney function, and End-stage kidney disease [RIFLE] or Acute Kidney Injury Network) or medical,^[15]^ or oncological (National Cancer Institute - Common Terminology Criteria for Adverse Events v5.0).^[1]^ By RIFLE criteria, AKI is defined as a sudden (1–7 days) and sustained (>24 hours) impairment of renal function. The RIFLE criteria define renal failure as having 4 stages. Furthermore, the RIFLE criteria provide a graded definition of AKI, which the staging criteria imply. Arbitrarily, AKI is divided according to the underlying cause into prerenal, renal, and postrenal types. In cancer patients, the same classification is followed and accepted by almost all the experts. Table 1 summarizes the common causes of AKI in malignancy.

**Table 1:** Common causes of acute kidney injury in cancer patients.

Prerenal
Extracellular fluid depletion (poor intake, vomiting, diarrhea, hypercalcemia), VOD, drugs such as Cal-I and NSAIDs, sepsis, and paraneoplastic
Renal (Intrinsic kidney damage)
* glomerular*
Membranous nephropathy (bronchogenic Ca, Lymphoma, myeloma)
Amyloidosis (MM, lymphoma)
Pamidronate-associated collapsing glomerulopathy (incidence unknown)
Light-chain deposition disease (MM, lymphoproliferative diseases)
*tubulointerstitial*
Acute tubular necrosis (toxic/ischemic)
Lymphomatous infiltration of the kidney
Light-chain deposition disease
Drugs (cisplatin, MTX, ifosfamide, allopurinol, rasburicase)
Lysozymuria
Disseminated intravascular coagulation
Nitroxide, inflammatory and proinflammatory
Intravenous contrast
Chemotherapy
Targeted and immunomodulating agents
Cast nephropathy (MM)
*vascular*
HUS (post-HSCT, gemcitabine, mitomycin C, etc.)
Tumor infiltration (renal cell carcinoma with renal vein thrombosis)
Postrenal (obstructive)
*Intratubular obstruction*
Uric acid nephropathy, crystallization, Ca-Ph product
Methotrexate
Cast nephropathy (MM)
Cyclophosphamide
e*xtrarenal obstruction*
Blockage of the drainage of urine, urinary bladder tumor, stones
Ureteral and urethral obstruction, primary urethral disease, retroperitoneal fibrosis (Ormond’s disease), and lymph node enlargement)
The common causes of obstructive uropathy are prostatic, bladder, uterine, and cervix cancer

Cal-I = calcineurin inhibitors, Ca-Ph = calcium-phosphate, HSCT = hematopoietic stem cell transplant, HUS = thrombotic-thrombocytopenic purpura/hemolytic-uremic syndrome, MM = multiple myeloma, MTX = methotrexate, NSAID = nonsteroidal anti-inflammatory, VOD = veno-occlusive disease.

### 3.2. Epidemiology of AKI in cancer patients

The annual AKI incidence is estimated at 13.3 million worldwide. Indeed, oncology patients, especially the elderly, have a higher chance of developing AKI during the first year after a cancer diagnosis. This combination negatively impacts patients’ survival.^[16]^ The prospect of higher mortality in a cancer patient who develops AKI, in addition to preexisting chronic renal conditions, is of grave concern and alarming to the patients. A bidirectional pattern describing the association between renal injury and cancer has been described.^[17]^ Indeed, AKI may affect pharmacokinetics, including the bioavailability of several anticancer medications and their safety profiles. Physicians unaware of these facts can result in suboptimal therapies or increased drug-induced renal toxicity risk.^[3]^ Furthermore, an overcautious approach may lead some physicians to omit certain highly effective anticancer medications that may cause AKI. Identifying, understanding, and managing every risk factor is vital in preventing AKI. Presently, there are no viable therapy schemes for AKI in malignancy; therefore, preventive measures should be highly prioritized.^[18]^

According to research, AKI is more likely to occur in cancer than in non-cancer patients.^[19]^ Cheng et al^[19]^ reported that AKI affected 7.5% of cancer patients. Approximately 1.6% of AKI cases were from the community, and 5.9% had AKI in hospitals. Studying 37,267 incident cases of cancer, the incidence of AKI was 258/1000 person-years, which decreased to 43/1000 person-years a year after a cancer diagnosis, and the 5-year risk was decreased further to 27%. The same study reported the highest 1-year risk (44%) in patients with renal cancer, 33% with hepatic cancer, and multiple myeloma (MM) (31.8%). Moreover, they reported that overall, and for most exact cancer sites, risks were high in patients with metastases at malignancy diagnosis.^[16]^ At 1 and 5 years, 4.5% and 7.6% of patients, respectively, had more severe AKI.^[16]^ Notably, the 28-day death rate of cancer patients on dialysis is projected to be 66% to 88%.^[20]^ AKI is very common in the inpatient setting; oncology, critical care, heart surgery, and transplantation units have extraordinarily high rates of AKI, exceeding 50%.^[21]^ Furthermore, recent research examined 163,071 patients undergoing systemic chemotherapy or targeted medicines for their disease and found that 6.6% had developed AKI. The AKI rate was 27/1000 persons/year with a total incidence of 9.3%. Myeloma [26%], bladder cancer (19%), and leukemia (15.4%) had the most significant 5-year AKI incidences. AKI occurs more with advanced cancer stages, CKD, and diabetes.^[22]^ The co-prescription of a diuretic and an angiotensin-converting enzyme inhibitor/angiotensin receptor blocker was associated with increased AKI rate in individuals aged 66 years and had systematic antitumor therapy.^[22]^ Another recent Chinese study found that 14% to 20% of cancer patients had AKI.^[22]^

### 3.3. Mechanism(s) of cancer-induced AKI

**Figure 1.**
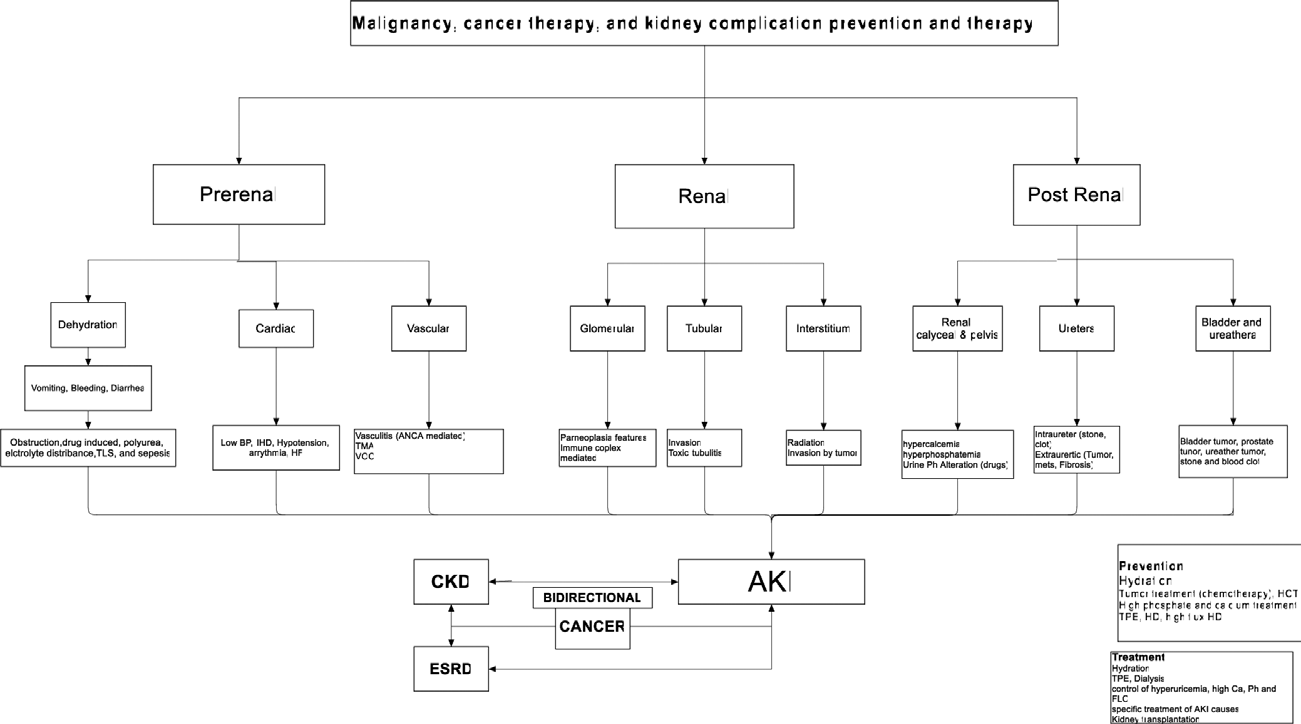
Mechanism(s) of Kidney complication, prevention, and treatment in cancer patients. AKI = acute kidney injury, ANCA = antineutrophil antibody, BP = blood pressure, Ca = calcium, CKD = chronic kidney disease, ESRD = end-stage renal disease, HF = heart failure, IHD = ischemic heart disease, Ph = phosphate, TLS = tumor lysis syndrome, VOD = veno-occlusive disease.

AKI mechanism(s) in cancer is multiple in origin, as will be discussed later. The possible mechanism(s) are summarized in Figure 1.

#### 1.3.3. Prerenal kidney causes of AKI.

Patient-related risk factors for AKI in cancer are similar to the other population; however, cancer patients have tumors, and their therapies as additional significant risk factors.^[23]^ A prospective multicenter study was conducted in 2013 to determine the primary risk variables for AKI. The study revealed that age, hypotension, sepsis, hypovolemia, preexisting CKD, concomitant vascular disease (including atherosclerosis), and congestive heart failure were potentially associated with AKI development.^[24]^ Additionally, vomiting and diarrhea due to some malignancies and chemotherapy increase dehydration rate and AKI. Prevention of AKI includes general strategies such as maintaining a good volume status, achieving hemodynamic stability, optimizing cardiac function, and treating anemia.^[21]^

#### 2.3.3. Intrinsic (renal) causes of AKI.

##### 2.1.3.3. Glomerular involvement.

Although glomerular disease in malignancy is generally uncommon, paraneoplastic glomerular involvement was reported.^[25,26]^ According to published studies, several types of carcinomas, and rarely leukemia and lymphoma, are related to membranous nephropathy. However, this relationship has been disputed, and registry data is only sometimes supportive.^[27,28]^ In 107 patients with biopsy-proven membranous nephropathy, 8.4% had underlying malignancy,^[29]^ and in prior publications, the incidence ranged from 5.8% to 10.6%.^[30,31]^ Because of this substantial connection, some experts recommend tumor screening in older individuals with membranous nephropathy.^[28,29]^ The exact mechanism by which cancer causes glomerular damage remains unknown. However, it may include tumor antigens being deposited in the subepithelial region, the creation of in situ immune complexes, and subsequent complement activation.^[32]^ This conception has some indirect support since treating the underlying tumor may result in clearing the nephrotic manifestations.^[33,34]^

With a frequency of 0.4% among 1700 studied individuals, the link between minimal-change disease and Hodgkin’s disease was observed, although rare.^[32]^ There are additional cases of membranoproliferative glomerulonephritis that progress quickly in conjunction with cancer.^[26]^ Pankhurst et al^[35]^ conducted retrospective case-control research comparing patients with antineutrophil cytoplasmic antibody (ANCA)-associated vasculitis with age- and gender-matched control to examine the relationship between ANCA vasculitis and cancer. One-third of the patients were diagnosed with malignancy when renal involvement was detected. Although cyclophosphamide treatment has been linked to bladder cancer (especially when taken daily), this research supports an earlier small series that demonstrated a direct connection between ANCA vasculitis and cancer, irrespective of the drug used to treat the vasculitis.^[36,37]^

##### 2.3.3.2. Renal parenchymal involvement.

Solid and hematological malignancies may affect the renal parenchyma; however, its clinical consequences are often unnoticeable. The most prevalent malignancies, besides multiple myeloma, are lymphomas and leukemias that affect the kidney, causing AKI.^[38]^ AKI, proteinuria, or hematuria may sometimes accompany lymphomatous invasion of renal tissues. However, this diagnosis is often accidental. Most patients have trivial kidney function disarrangement; however, AKI occurs mainly due to infection, aminoglycoside, diuretics, hypercalcemia, TLS, and chemotherapy rather than lymphoma kidney invasion.^[39]^ Kidney involvement was suspected before the kidney biopsy, mainly based on radiographic evidence of enlarged kidneys in 80% of B-cell lymphoma.^[40]^ Most patients show bilateral enlargement on renal imaging. AKI coupled with lymphomatous infiltration may respond effectively to treatment focused on the underlying malignancy. Recently published data reported that AKI occurred in 29% of B-cell lymphoproliferative disorders.^[41]^

Tumor cells that compress tubules and disturb renal microcirculation are likely to be the etiology of AKI.^[42]^ In these situations, AKI is often quickly reversible with prompt treatment. When red blood cell casts, hematuria, or proteinuria are discovered, cancer-related glomerulonephritis, such as membranoproliferative glomerulonephritis or amyloidosis, should be considered.^[43]^ In these situations, a kidney biopsy is essential for determining the diagnosis prognostication and guiding the best course of therapy. Malignancy-related glomerulonephritis may be the first sign of cancer and is relatively rare; therefore, it should be suspected, and age-appropriate cancer screening must be effectively conducted.^[44]^ Significantly, lymphomatous kidney invasion causes AKI, which improves with anticancer treatment.^[18]^ Rarely extrarenal solid metastatic tumors can lead to AKI. There are a few examples of AKI caused by solid tumor infiltration; when they do, there is often extensive parenchymal invasion by the tumor. The most common solid tumors that spread to the kidney are bronchogenic carcinomas, followed by stomach and breast carcinomas.^[45]^

The cellular damage and AKI result from the filtration of lysozyme at the glomerulus and its uptake by proximal tubular cells. Patients with acute promyelocytic, monocytic, or chronic myelomonocytic leukemia, in which malignant cells create excessive levels of lysozyme, are more likely to develop lysozymuria, a rare condition.^[18]^ In these circumstances, urine protein electrophoresis may contain a significant amount of lysozyme.

Patients with MM are more susceptible to AKI. The etiology of AKI in these individuals is complex and varies. AKI is quite typical and may make the course of myeloma more severe in 20% to 50% of patients.^[18]^ AKI caused by light chain cast nephropathy is regarded as a myeloma-defining event according to the 2014 International Myeloma Working Group criteria.^[33]^ Rarely, additional hematological malignancies such as Waldenstrom macroglobulinemia, chronic lymphocytic leukemia, or lymphoma may be linked to light chain cast nephropathy.^[46]^

The thick ascending limb of the loop of Henle is where Tamm-Horsfall protein (uromodulin) binds to free light chain (FLCs), freely filtered by the glomerulus, to form insoluble casts that impede the tubular lumen and cause local inflammation.^[34]^ The carbohydrate moieties on Tamm-Horsfall proteins engage non-covalently with shared binding sites on the kappa and lambda light chains.^[47]^ Reduced tubular flow rates and increased urine electrolyte concentrations (such as those associated with diuretics) encourage these interactions and the development of obstructive casts.^[48,49]^ Additionally, there is a clear connection between serum FLC concentration and the risk of cast nephropathy.^[50]^ Diffuse interstitial inflammation is observed upon histological analysis; the leakage of light chains may have brought this inflammation into the renal interstitium. Due to this interstitium infiltration, different pro-inflammatory pathways are activated, and continued inflammation may lead to irreversible kidney damage, emphasizing the need for prompt and aggressive therapy.^[51]^ However, new research projects are needed to explore inflammatory and proinflammatory factors’ role in AKI in malignancy.

Serum FLC measurements, including quantitative measurements of kappa and lambda FLCs and serum and urine protein electrophoresis, are the main components of cast nephropathy diagnosis.^[52]^ To differentiate cast nephropathy, where proteinuria is mostly unbound albumin FLCs, from paraprotein-related glomerular disorders, such as albuminuria and serum FLCs, serum protein, and urine protein electrophoresis are used.^[53]^ According to a Mayo Clinic study, the median amount of urinary albumin excretion in individuals with light-chain cast nephropathy was 7%, and urinary albumin excretion was <25% of total urinary protein excretion.^[54]^ Kidney biopsy is advisable when urine albumin excretion is high, especially if blood FLC levels are <50 mg/dL.^[33]^

During the last 10 years, cast nephropathy treatment has advanced significantly. It now focuses on providing appropriate hydration to increase tubular flow and treats preexisting volume depletion (“flushing out the tubular casts”), as the chemotherapy quickly lowers FLC levels. Proteasome inhibitors such as bortezomib are combined with other medications such as thalidomide, corticosteroids, vincristine, and Adriamycin.^[49,55]^ These regimens were linked to dramatically increased survival high and renal function improvement rates.^[55,56]^ Importantly, within a median duration of r 1.34 months, bortezomib works swiftly to enhance GFR.^[57]^ Adding alkylating medication (bendamustine) to a prednisone and bortezomib regimen increased kidney response rates to more than 80%, with most of the response happening within 6 weeks.^[49,58]^

There is still interest in using RRT to quickly remove FLCs when more decisive chemotherapy is used because a rapid drop in the blood concentration of FLCs is essential for restoring kidney function. A continuous debate is ongoing over therapeutic plasma exchange (TPE) and high-cutoff hemodialysis (using large-pore dialysis membranes to enhance FLC removal). Randomized controlled studies on plasmapheresis have been limited and inconclusive in 97 participants.^[59]^ Two recent experiments have been reported, and most recently, the use of high flux dialyzers with better capacity for light chain removal has been examined. There was no advantage of high flux dialysis over standard dialysis in a European study of FLC elimination by prolonged hemodialysis.^[60]^ However, in Patients With MM and Renal Failure Due to Myeloma Cast Nephropathy study that included patients with renal biopsy-proven cast nephropathy revealed that high-flux dialysis was advantageous.^[61]^ In this experiment, high-flux dialysis increased dialysis dependency to 56.5% compared with conventional dialysis (35.4%, *P* = .04). Due to the inconsistent results of the two studies, it is still being determined whether high-cutoff hemodialysis is beneficial or not.^[62]^ Therefore, new research is required to elucidate the issue further.

#### 3.3.3. Obstructive uropathy (post-renal).

More often than the general population, AKI’s postrenal causes should always be considered in cancer patients. Urogenital system obstruction is prevalent more with uterine, prostate, and cervical tumors.^[63]^ Urinary system obstruction by lithiasis occurs in patients with malignancy due to stone formation or clots. Ureter obstruction occurs because of stones or compression.^[64]^ Urinary bladder outlet obstruction and tumors have been reported to cause obstructive uropathy. Penile and vaginal tumors rarely lead to obstructive uropathy. The absence of hydronephrosis does not exclude obstructive uropathy because pelvic-ureteric dilatation may not develop if a retroperitoneal tumor or fibrosis significantly encases the kidney pelvises and ureters. Retroperitoneal fibrosis (a cancer complication) rarely occurs and is often idiopathic. However, it occurs mainly as a radiotherapy complication for malignant pelvic organ cancers and lymphoma patients.^[65]^

## 4. Cancer-induced AKI prevention

Beyond TLS, no specific preventive measures should be implemented in cases of tumor-specific AKI. Preventing AKI in cancer includes adequate hydration, prophylactic antibiotics, and hematopoietic growth factors in patients with neutropenia/febrile neutropenia. Furthermore, avoiding the repeated and frequent use of potentially nephrotoxic drugs and contrast agents is essential to prevent AKI.

## 5. Cancer complications cause renal injury

### 5.1. Thrombotic microangiopathy

According to previous prospective research, almost 6% of patients with metastatic cancer developed microangiopathic hemolytic anemia.^[66]^ The pathological traits of TMA include endothelial edema, microvascular blockage, and intrarenal or systemic microvascular thrombi.^[67]^ It is typically associated with renal failure, neurological problems, and gastrointestinal complaints.^[35,36]^

It is well-documented that cancer affects the kidneys and causes AKI, CKD, and other kidney dysfunctions. TMA syndrome is highly correlated with the use of anticancer medications. One common causative drug is mitomycin C, with a 2% to 10% risk that has risen considerably with a collective dose of 40 mg/m^2^.^[68]^ Cisplatin, 5-fluorouracil, and bleomycin have been linked less commonly to TMA.^[11]^ Treating cancers such as pancreatic, bladder, and advanced non-small-cell lung malignancies with the nucleoside analog gemcitabine is common. The cumulative incidence was 0.31%, greater than the estimated 0.01%.^[69]^ TMA syndromes and hypertension (HTN) are linked, and HTN severity in TMA correlates with poor outcomes.^[70]^ Glomerular ischemia caused by the microvascular capillary blockage in the glomeruli and other kidney tissues is the most likely mechanism through which TMA causes HTN.^[71]^ Patients who take gemcitabine have a risk of developing new or worsening HTN. Hence, regular weekly follow-up at the start of treatment is required, enabling the early detection of TMA and AKI linked to gemcitabine.^[69]^

### 5.2. Tumor lysis syndrome

Hematological malignancies or, less commonly, solid tumors may cause a potentially fatal condition known as TLS. It happens due to the tumor cells being massively destroyed at the start of cancer chemotherapy, but it may also occur spontaneously in one-third of cases.^[72]^ According to the consensus definition, the characteristics of biological TLS include the rapid release of intracellular contents with a subsequent 25% change in the level above average for any two or more serum values of uric acid, potassium, phosphate, and calcium within 3 days before or 7 days after the start of chemotherapy.^[73,74]^ Additionally, a significant amount of purine released from DNA catabolism is transformed into xanthine and uric acid, increasing the risk of intrarenal and extrarenal obstructive uropathy. Acute renal damage is detrimental to complete remission and survival rates in this situation.^[75,76]^ Uric acid, xanthine, and calcium phosphate crystal precipitation in renal tubules are one of the main mechanisms of TLS-induced AKI.^[77]^ Additional mechanisms may also contribute to acute tubular necrosis, including the increase in pro-inflammatory cytokines production, affecting renal microcirculation, and increasing renal parenchyma tumor infiltration. According to the consensus definition, other clinical TLS signs, including cardiac arrhythmia and seizures, are also features.^[73]^ TLS may sometimes resemble sepsis and present with acute respiratory failure, hemodynamic instability, and systemic inflammatory response syndrome. Abdel-Nabey et al^[78]^ reported that in 153 TLS patients admitted into intensive care, AKI was reported in 82% of the patients.

A recombinant urate oxidase enzyme (rasburicase) administration is linked to increased remission rates for underlying cancers.^[79]^ Furthermore, the urate-oxidase enzyme levels may indirectly indicate the liability for high tumor cell lysis and chemosensitivity. It was reported that delayed hyperuricemia treatment might have long-term effects.^[78]^ Identifying high-risk TLS patients as soon as possible to prevent TLS and TLS-induced AKI is crucial.^[80]^ The cornerstone of TLS care is hydration and hypouricemic medications (allopurinol and rasburicase). Recombinant urate oxidase (rasburicase) degrades uric acid to ten times soluble metabolites.^[79]^ Despite rasburicase’s widespread use, Darmon et al^[72]^ reported that TLS occurred in >60% of HR-TLS patients, and half also had AKI. Additionally, only a limited number of patients were included, and most of those studies did not involve intensive care unit admitted patients. Therefore, more data is needed on the overall care and result of TLS among adults, particularly with rasburicase use.

The exact incidence of TLS is unknown. Factors such as tumor load, tumors with a high proliferation rate, tumors with increased sensitivity to chemotherapy, and previous renal illness or impairment may all raise the occurrence of TLS. Race and gender are not essential reported factors in the propensity to develop TLS. According to the documented National Inpatient Sample database, non-Hodgkin lymphoma (30%), solid tumors (20%), acute myeloid leukemia (19%), and acute lymphocytic leukemia (13%) are the most frequent malignancies linked to TLS. TLS causes around 21% of the total in-hospital mortality.^[81]^

#### 1.5.2. AKI pathogenesis in TLS.

Severe tumor cell lysis contributes to the pathophysiology of AKI due to significant quantities of calcium, phosphate, and uric acid release. The kidney ordinarily excretes these substances; preexisting renal impairment worsens TLS’s metabolic disturbances. AKI may result from the buildup of uric acid-calcium phosphate crystals in the renal tubules, which may sometimes worsen by concurrent intravascular volume depletion. The kidney is the main organ for uric acid, calcium, potassium, and phosphate clearance. Patients with volume depletion or renal impairment are more likely to develop acute kidney damage in TLS status. The AKI has a complex etiology and is often oliguric in TLS. The primary cause of AKI, however, is uric acid nephropathy. Patients at high or intermediate risk and those with established TLS should undergo comprehensive medical treatment in a hospital and sometimes an intensive care unit. Patients with refractory AKI in TLS mostly need RRT.^[81]^

### 5.3. Veno-occlusive disease

Despite initial renal problems in the early post-transplantation stages, hepatorenal syndrome (HRS) is the most prevalent cause of severe AKI after a hematopoietic stem cell transplant (HSCT). Depending on the diagnostic criteria used, the incidence of VOD might vary from 5 to 70% in various publications.^[82]^ VOD is regarded as a conditioning-related hazard most often linked to cyclophosphamide, busulfan, and total-body irradiation regimens.^[83]^ Older patients, female gender, advanced malignancy, prior abdominal radiation, amphotericin B, vancomycin, or acyclovir medication are additional factors for developing VOD.^[84]^

Jaundice, tender liver enlargement, and weight gain are all clinical signs of VOD. Acute hepatic graft-versus-host disease (GVHD), sepsis, drug-induced cholestasis, calcineurin inhibitor toxicity, gall bladder disease, and use of total parenteral nutrition are a few disorders that might resemble VOD, making the differential diagnosis list long and differentiate difficultly.^[84]^ Symptom-onset timing helps establish the diagnosis; VOD often manifests during the first 30 days after HSCT. Sodium retention is the primary feature in the early stages of HRS, followed by weight gain, edema, and ascites that follow right upper quadrant pain and jaundice. AKI affects around 50% of VOD patients; however, almost all patients have some degree of renal insufficiency.^[85]^ Although diuresis or analgesia for right upper quadrant pain may be necessary for mild illness, nephrotoxic analgesics are not advisable. In HSCT, the mortality rate for severe VOD is very high.^[86]^

The pathogenesis of VOD is complex. It is primarily due to injury of the blood vessels’ endothelium, mainly in the liver sinusoids, leading to endothelial cell detachment with downstream embolization and obstruction.^[87]^ Several factors are implicated in endothelial damage, such as conditioning regimens,^[88]^ cytokines,^[89]^ endogenous microbial products,^[90]^ drugs used during the transplant (such as granulocyte colony-stimulating factor or calcineurin inhibitors),^[91]^ and the engraftment process itself.^[92]^ The increased risk of VOD linked to greater plasma levels of cytotoxic medicines such as busulfan and cyclophosphamide demonstrates that conditioning regimens play a significant role in the etiology of VOD.^[93]^ The cytochrome P450 complex metabolizes chemotherapy, creating toxic compounds that are eventually transformed into nontoxic ones by the glutathione enzymatic system and then can be excreted.^[94]^ Decreased detoxifying capacity owing to an immature enzymatic system should, at least in part, explain the greater frequency of VOD in children.^[95]^ Moreover, endothelial cell detachment is possibly connected to nitric oxide deficiency, resulting from postconditioning damage.^[96]^ Blood flow blockage is increased by the growth of perisinusoidal cells and subendothelial fibroblasts in the liver.^[97]^ VOD is currently categorized as a transplant-related endothelial dysfunction due to the significant involvement of endothelial damage in its pathophysiology, along with posttransplant microangiopathy, idiopathic pneumonia, diffuse alveolar hemorrhage, and engraftment syndrome.^[86]^

Numerous insults may cause the development of HSCT-associated AKI, some of which are unique to this clinical situation and others more widespread, such as sepsis. Hepatic VOD during HSCT, an independent risk factor for AKI, may result from liver damage sustained during the procedure (particularly during the induction phase).^[98]^ Although the typical incidence of VOD is 13.7%, newer therapy regimens have dramatically reduced the disease percentage.^[98]^ Damage to sinusoidal endothelial cells and hepatic cells brought on by cytoreductive regimens causes a hepatic sinusoidal blockage.^[98]^ Hepatic VOD resembles HRS and is marked by jaundice, oliguria, and ascites. In these circumstances, hypervolemia is often resistant to diuretics, and spontaneous recovery is uncommon. AKI negatively impacts survival, with a higher death rate in patients needing RRT in 80% of these patients. Prostaglandin-E, pentoxifylline, and low-dose heparin are used for preventing and treating VOD.^[99]^

Although hepatic microangiopathy is the hallmark of VOD histologically, this change is not seen in the kidney histology at autopsy,^[90]^ which supports the theory that the renal damage in HRS is hemodynamic rather than structural. The coagulation cascade may be a site of intervention in this condition since the endothelial injury has been postulated to play a significant role in the onset of VOD. Defibrotide is a single-stranded polydeoxyribonucleotide. It has been proven for use in renal or pulmonary dysfunction following HSCT.^[100]^ Defibrotide (an antithrombotic and fibrinolytic drug) seems advantageous if started early and may enhance GFR.^[101]^ It has shown promising effects in recent controlled studies after unsatisfactory findings from earlier investigations of antithrombotic and thrombolytic drugs.^[101]^ Defibrotide contains fibrinolytic, antithrombotic, and anti-ischemic characteristics in addition to binding to vascular endothelium. Prospective studies are being conducted to verify its effectiveness in preventing and treating VOC.^[87]^

## 6. Cancer therapy-induced AKI

### 6.1. Hematopoietic stem cell transplantation

HSCT is a critical and potentially curative therapy for cancer patients, particularly those with hematological malignancies. The conditional use of chemotherapy, radiation exposure, sepsis, VOD, TMA, GVHD, or nephrotoxic drugs may cause AKI, making HSCT more challenging.^[102,103]^ Whether an allogeneic or autologous transplant is carried out and high-dose or low-intensity chemotherapeutic precondition regimens are used, the incidence of HSCT-associated AKI varies from 15% to 73%.^[102]^ A greater risk of AKI is linked to allogeneic HSCT and myeloablative regimens.^[103]^ RRT was required in only 5% of patients; the number surpassed 30% in high-risk individuals.^[103]^ AKI is linked to a higher mortality rate, occurring before engraftment and early in the post-HSCT course.^[104]^ Since post-HSCT-AKI often affects people with severe immunosuppression and may be accompanied by additional conditions such as GVHD, sepsis, and other serious illnesses, which may be a challenge to treat. The relationship between the incidence of AKI post-HSCT and the ultimate onset of CKD has been documented in the literature. Hingorani reported that CKD might develop between 6 months and ten years following HSCT, with a cumulative incidence of 7% to 48%. AKI, acute and chronic GVHD, older age at HSCT, a decline in baseline GFR, HTN, use of calcineurin inhibitors, and exposure to whole-body irradiation are risk factors for developing CKD following HSCT.^[105]^

Patients with HSCT-associated TMA may ultimately get CKD or AKI.^[106]^ Endothelial edema, destruction, and fibrin thrombi inside capillary loops and arterioles are the hallmarks of the lesion.^[63,64]^ The conditioning programs for HSCT can potentially result in renal endothelial damage and TMA. Due to direct endothelial cell damage calcineurin inhibitors use, GVHD may exacerbate TMA.^[107]^ Treatment options include stopping or lowering the dosage of calcineurin inhibitors, TPE, and defibrotide.^[108]^ TPE response rates have been found to range between 27% and 80%, including 64% for TPE with cyclosporine cessation.^[109]^ Further evidence is required to support the use of rituximab for TMA post-HSCT.^[43,109]^

Another important risk factor for the development of AKI in HSCT patients is acute GVHD.^[110]^ Cytokine-mediated renal inflammation and cyclosporine use can cause AKI. Other causes of AKI in patients with GVHD include viral reactivation (cytomegalovirus), vomiting, and diarrhea, which may be severe, inducing prerenal AKI. Prednisone, antithymocyte globulin, sirolimus, and mycophenolate mofetil are all used in graft versus host disease therapy and supportive measures.^[111]^

## 7. Anticancer agent-induced-AKI

Generally, 3 chemotherapy medication types are used to treat cancer: traditional, targeted, and immunotherapies.^[105,106]^ The tubules, interstitium, vasculature, and glomeruli may all sustain damage from conventional chemotherapeutic drugs, resulting in different types of AKI. Acute tubular injury (ATI), acute interstitial necrosis (AIN), and other glomerular and vascular injuries are the leading causes of anticancer-induced AKI.^[104]^ The pathogenesis of AKI or kidney involvement differs from one drug to another.

### 7.1. Standard agents

TMA’s microvascular process complicates gemcitabine, mitomycin C, and cisplatin treatment.^[17,112,113]^ Drug-induced endothelial damage with the release of von Willibrand factor and plasminogen activator inhibitor, as well as exposure of the endothelial surface to fibrin and platelets, all aid the damage process inside the renal microvasculature.^[114]^ Even if drug withdrawal is necessary, treatment using various methods has proved unsuccessful. Some case studies have reported Eculizumab and rituximab, and TPE is often ineffective but still can be tried.^[115]^

Pamidronate and zoledronate have been used less often to treat glomerular damage, especially podocyte injury with the histological subtypes of focal segmental glomerulosclerosis or minimal change disease.^[116]^ In most of these situations, severe proteinuria is linked to the gradual emergence of renal failure over an extended period. ATI is the most frequent lesion associated with AKI with conventional chemotherapy, and many medications may produce ATI.^[113,117]^ Ifosfamide, pemetrexed, zoledronate, and others damage the tubular epithelium cells by direct intoxication, inducing apoptosis, causing oxidative stress, and mitochondria injury.^[118]^

Crystalline-induced AKI has been linked to methotrexate.^[113,117]^ AKI is facilitated by intratubular crystal precipitation in conjunction with obstructive and inflammatory interstitial damage. While urinary alkalinization and intravenous fluids are used for prophylaxis and therapy, severe toxicity may need hemodialysis and glucarbidase.^[119]^ Methotrexate clearance is primarily by the kidneys. Toxic amounts of methotrexate may build up in the presence of AKI, resulting in severe bone marrow toxicity. Therefore, AKI brought on by methotrexate is a medical emergency. Physicians should actively evaluate GFR in such cases and consider using hemodialysis or glucarbidase early on if methotrexate levels increase. Lastly, chemotherapy drugs, such as carboplatin, ifosfamide, and adriamycin, may result in AKI from interstitial nephritis.^[118]^

### 7.2. Targeted agents

The Ras-Raf-MEK-ERK signal transduction cascade is perhaps the most significant pathway in human cancer.^[120]^ The oncogenesis signaling cascades promote tumor development by explicitly targeting the gene mutations that characterize individual tumors.^[121]^ Unfortunately, medications that block this cascade are associated with AKI, proteinuria, and HTN,^[121]^ despite their effectiveness in treating cancer. This often occurs because the mechanisms involved in angiogenesis may serve similar changes in the kidney. Anti-angiogenesis medications that target vascular endothelial growth factors, such as bevacizumab, axitinitib, sorafenib, and sunitinib, predominantly induce AKI. At the same time, focal segmental glomerulosclerosis and AIN have also been seen with severe HTN.^[121]^ Vemurafenib and dabrafenib, serine/threonine kinase BRAF (BRAF is “v-RAF murine sarcoma viral oncogene homolog B1”) inhibitors, have been associated with dose-related AKI.^[122]^ Although there is little histological information available,^[122]^ it is likely that the suppression of the mitogen-activated protein kinase pathway causes acute tubular necrosis damage. Most of the time, stopping a drug is linked to the reversal of AKI. Anaplastic lymphoma kinase inhibitor crizotinib, which induces AKI via tubulointerstitial damage, is only partly curable by stopping the treatment.^[121]^ Since this is a developing field, doctors should be aware of medication toxicity in patients with unexplained AKI and consider doing a kidney biopsy to guide them for further actions. This is a growing research field, and other studies are required.

### 7.3. Immunotherapies

The well-known causes of AKI include older drugs like interferon and high-dose interleukin (IL)-2.^[123]^ Although the cytokines IL-6 and -13 may also be involved, natural interferon binding to podocyte receptors and modification of normal cellular proliferation may enhance podocyte damage.^[123]^ High-grade proteinuria from focal segmental glomerulosclerosis or minimal change nephropathy is a common clinical symptom of interferon-associated AKI. Drug stoppage (with or without steroids) may reverse AKI and proteinuria with little-to-no change in the illness progression, but it works less well in segmental glomerulosclerosis.

Ipilimumab, nivolumab, and pembrolizumab are immune checkpoint inhibitors that improve tumor killing by inhibiting dendritic cells and tumor antigen ligands from attaching to CTLA-4 and PD-1 receptors, respectively.^[124]^ As a result, the T-cell destruction of tumor cells is activated and increased. Unfortunately, AIN and a range of glomerular lesions are caused by a loss of tolerance to drugs that tend to trigger an immune response.^[124]^ In general, drug withdrawal combined with steroids helps to correct AKI, particularly if this action is initiated early, although a significant proportion of patients develop CKD. However, it is crucial to understand that AKI, in the presence of immune checkpoint inhibitors, may be linked to tubular or glomerular damage, necessitating a kidney biopsy to confirm it. Host cells are extracted and modified to express receptors that identify and bind tumor antigens to create chimeric antigen receptor T-cells.^[125]^ Cancer cells are specifically targeted and eliminated by T lymphocytes. However, this approach encourages cytokine release syndrome and macrophage activation, which may cause a capillary leak and prerenal AKI.^[126]^ To lessen the tumor load, steroids and prior chemotherapy are used to prevent and treat AKI.^[126]^ An IL-6 receptor blocker and steroid may diminish side effects due to severe cytokine release syndrome.^[126]^

## 8. Cancer-induced electrolytes abnormalities and kidney involvement

### 8.1. Hyperuricemia

Hyperuricemia usually develops due to the fast degradation of intracellular nucleic acids released after the lysis of cancer cells during 48 to 72 hours following starting anticancer therapy.^[127]^ Over 99% of uric acid is ionized at physiological pH.^[128]^ However, the uric acid may crystallize at the tubular level in the presence of an acidic pH and a high concentration, causing the blockage and leading to acute obstructive uropathy. Clinical symptoms include hematuria, flank discomfort, HTN, hyperazotemia, acidosis, edema, oliguria, anuria, lethargy, and sleepiness.^[129]^ In the absence of conclusive evidence, in patients with hyperuricemia (uric acid > 8 mg/dL), elevated lactate dehydrogenase (>2 folds of the upper limits of normal), despite adequate volume resuscitation without evidence of post-obstructive cause, acute oliguric or anuric kidney failure should be sought as potential reasons.

All hematological cancer patients receiving chemotherapy are advised to receive TLS prophylaxis. Additionally, prophylaxis is recommended for all high- and moderate-risk patients, including those with significant tumor loads, decreased GFR, and extremely chemosensitive malignancies. The precise prophylaxis plan should be customized to the patient’s clinical situation. It should include lowering uric acid levels, maintaining appropriate hydration and tubular urine flow rate, and managing abnormal electrolyte levels, especially hypercalcemia. Hydration improves renal blood flow, maintains GFR, and lowers extracellular uric acid concentrations to prevent urate nephropathy. Intravenous fluid administration (≥3 L/d) and xanthine oxidase inhibitors in high-risk patients before chemotherapy are two methods for preventing and treating renal damage due to hyperuricemia, especially in TLS.^[75]^

Allopurinol, an isomer of hypoxanthine, prevents the production of uric acid by blocking the enzyme xanthine oxidase. Allopurinol will raise plasma levels of the uric acid precursors hypoxanthine and xanthine. These metabolites in alkaline urine may crystallize and deposit in the kidneys and cause xanthine nephropathy. Fever, rash, eosinophilia, systemic hypersensitivity responses, Stevens-Johnson syndrome, hepatitis, AIN, and bone marrow suppression are known side effects of allopurinol. Allopurinol should be started 2 to 3 days before the definitive treatment and continued for 10 to 14 days. It is crucial to understand that xanthine oxidase inhibitors can only prevent new uric acid elevations because they cannot reduce the existing high uric acid level. A non-purine analog xanthine oxidase inhibitor called febuxostat is helpful for people who cannot take allopurinol. In a recent study, febuxostat was shown to be better than allopurinol in decreasing uric acid levels. However, the clinical outcome was similar.^[130]^

Recombinant urate oxidase (rasburicase) may treat hyperuricemia in individuals with both underlying renal impairment, AKI or CKD, and hyperuricemia at diagnosis. Rasburicase catalyzes the conversion of soluble allantoin that is quickly eliminated by the kidney. The medication causes a drop-in blood uric acid levels due to its rapid onset of action (within 4 hours). In leukemia, lymphoma, and solid tumor malignancies undergoing anticancer therapy likely to cause tumor lysis and rise of plasma uric acid, rasburicase is suggested as a single agent for high uric acid treatment with hydration. Rasburicase is not advised for use in individuals with glucose-6-phosphate dehydrogenase deficiency because it produces toxic hydrogen peroxide while converting uric acid to allantoin.^[131]^ Since the discovery of rasburicase, hemodialysis for treating TLS is probably no longer necessary. Hemodialysis is still a successful treatment option for electrolyte and acid-base problems, mainly when oliguric AKI is present. Continuous renal replacement treatments prevent “rebound” metabolic abnormalities, although higher clearance values (at least 30–40 mL/kg/h) should be sought if a continuous renal replacement is used.

The enzyme uricase or urate oxidase, which catalyzes the conversion of uric acid to the more water-soluble allantoin, is a novel strategy for preventing and treating uric acid nephropathy.^[132]^ Since 1975, a nonrecombinant urate oxidase formulation has been used in Europe; however, there has been a 5% rate of allergic responses.^[133,134]^ Rasburicase, a recombinant uricase preparation, has been approved by the Food and Drug Administration to treat pediatric patients at risk for TLS. The recombinant rasburicase has fewer allergic responses and is both efficient and well-tolerated. In patients at risk for tumor lysis, Goldman et al^[135]^ demonstrated a decreased area under the curve for uric acid over 96 hours with 128 ± 70 mg/dL in the Rasburicase group versus 329 ± 129 mg/dL in the allopurinol group (*P* = .001). Hemodialysis for the AKI was required for 1 patient in the allopurinol group but for none in the rasburicase group.^[135]^ Other research reported that 3-day rasburicase therapy reduced plasma uric acid levels in 49 hyperuricemia adult patients from a median of 11.9 to 0.7 mg/dL.^[129]^ One hundred adult patients with aggressive non-Hodgkin lymphoma who received a preventive regimen of 0.2 mg/kg intravenous Rasburicase starting the day before chemotherapy and intravenous hydration were studied.^[136]^ Within 4 hours, the serum uric acid levels for all the patients were controlled, and throughout the observation period, none of the patients had hyperuricemia, and none of them required hemodialysis or had a rise in serum creatinine. It has been reported that urate oxidase is filtered through the glomeruli and may degrade the precipitated uric acid crystals and alleviate the intratubular blockage.^[137]^ Further research is required to understand Rasburicase’s function in treating already occurred uric acid nephropathy.

### 8.2. Phosphate, calcium, and potassium abnormalities

Hyperphosphatemia and hypercalcemia cause calcium-phosphate crystals to precipitate into the renal tubule. The danger of calcium phosphate deposition in the kidney and other organs increases when the calcium-phosphorus product is >70 mg^2^/dL, leading to metastatic calcifications.^[138]^ Particularly at the renal level, it is possible to detect clinical disorders ranging from acute obstructive uropathy to nephron calcinosis and lithiasis using a computed tomography scan.^[139]^ A prevalent cause of hypercalcemia related-AKI occurs in 20% to 30% of all malignancies, including commonly MM and squamous cell carcinomas.^[137]^

Direct renal vasoconstriction is thought to mediate the reversible reduction of GFR in severe hypercalcemia. This was shown in research comparing the GFR and renal blood flow in rats with hypercalcemia with rats with normocalcemia.^[140]^ Additionally, volume loss may happen due to various processes affecting renal function. A prime mechanism is a deficiency in urine concentrating capacity, which results in polyuria and polydipsia. The downregulation of aquaporin 2 (AQP2) water channels in the collecting tubules^[141]^ and tubulointerstitial damage brought on by calcium buildup in the medulla are suggested to be the causes.^[142]^ Research has revealed a substantial decrease in AQP2 expression in the collecting ducts of hypercalcemic rats as compared to controls confirmed the dysregulation of AQP2; however, the molecular mechanism is unclear.^[141]^ Additionally, calcium reabsorption is decreased by activating the calcium-detecting receptors found on the basolateral membrane of the thick ascending limb of the Henle loop.^[143]^ The sodium-potassium-chloride cotransporter in the luminal membrane of the thick ascending limb of the loop of Henle is inhibited due to calcium-sensing receptor activation, which decreases sodium chloride reabsorption and increases urine calcium excretion. Additionally, it is believed that the production of prostaglandin E2 by hypercalcemia decreases sodium chloride reabsorption in the loop of Henle.^[143]^ AKI brought on by hypercalcemia is often curable by reducing blood calcium levels and volume expansion with a saline solution. Rarely, severe hypercalcemia may lead to AKI by intratubular calcium-phosphate deposition, mainly if the calcium-phosphate concentration product is ≥70 mg^2^/dL. It should be noted that nephrocalcinosis-associated AKI may not get better with therapy for hypercalcemia; instead, patients may end up with substantial CKD, depending on how long the hypercalcemia has lasted. This emphasizes the need for a rapid decrease in serum calcium levels to prevent CKD.

Hypotension, dry mucous membranes, flat neck veins, weakness, disorientation, nausea, and polyuria are symptoms of hypercalcemia. Intravascular volume resuscitation, improving GFR, increasing calcium excretion, and hypercalcemia-lowering agents are all needed to reduce the risk of AKI due to hypercalcemia.^[137]^ Low-dose pamidronate (typically 60 mg or lower) infused over 4 hours effectively decreases serum calcium over several days and has a longer-lasting effect of preventing recurrent hypercalcemia in addition to aggressive intravenous fluids with normal saline, even without diuretics, unless hypervolemia is present.^[137]^ In rare cases, severe hypercalcemia in AKI may need dialysis.^[137]^ RANKL stands for receptor activator of nuclear factor kappa-beta ligand. It is also known as osteoprotegerin ligand, osteoclast differentiation factor, or tumor-necrosis-factor-related activation-induced cytokine.^[144]^ The primary function of RANK is to preserve bone physiologic remodeling.^[144]^ Denosumab, a humanized monoclonal antibody against RANK ligand, does not need dosage adjustment for GFR^[145]^ and can effectively reduce high serum calcium.

Hyperphosphatemia might happen within 24 to 48 hours following the initiation of chemotherapy. Nausea, vomiting, diarrhea, lethargy, and seizures are usual presenting symptoms.^[127]^ Reported data showed that cancer cells might have up to 4 times more organic and inorganic phosphorus than nonmalignant cells. Additionally, acute renal insufficiency may significantly exacerbate this imbalance, especially when it is linked to uric acid precipitation or other side effects chemotherapy brings.^[138]^

Cardiac arrhythmias are a dangerous complication of high serum potassium levels. Cellular lysis causes a significant release of potassium into the extracellular space. It might happen 6 to 72 hours after the start of chemotherapy.^[128]^ AKI, CKD, acidosis, or excessive iatrogenic potassium administration during the induction phase of chemotherapy are a few concomitant conditions that may exacerbate this hyperkalemia.

## 9. AKI outcome in cancer patients

AKI is a frequent complication in cancer patients linked to decreased treatment dosage intensity, worse remission rates, higher mortality, hospitalization duration, and higher healthcare cost. The patients with AKI and cancer have in-hospital mortality of 12%, whereas this figure is only 0.9% for the AKI patients without cancer. In two other recent studies, AKI was reported in 11% and 20% of cancer patients over a year of follow-up, with hematological cancer patients at greater risk.^[16,20]^ However, depending on the severity of AKI and underlying comorbidities, investigations in higher-risk, critically sick populations have shown that 8% to 60% of patients needed RRT.^[146]^ Within a year after AKI, 5% of patients in a large Danish cohort with cancer and AKI needed RRT.^[16]^ Early detection and good hydration are crucial methods to prevent AKI in cancer.

## 10. Chronic kidney disease

CKD is a significant long-term side effect of HSCT, especially allogeneic HSCT (which develops in 15–20% of survivors).^[147]^ The overall prevalence of CKD in allogeneic HSCT survivors constitutes a future public health issue due to the increased number of allogeneic transplants.^[148]^ A low-grade renal TMA is assumed to be the primary cause of most CKD patients after HSCT.^[148]^ These patients usually have persistently increased plasma creatinine, HTN, and anemia. Proteinuria and hematuria are recognized features. There are no characteristic renal imaging features; however, a kidney ultrasound is usually advisable. Kidney biopsy results are unlikely to alter the prognosis of CKD significantly, and the biopsy carries increased bleeding risks in patients with thrombocytopenia and coagulopathy. Renal histological findings include mesangial layer damage, basement membrane duplication, glomerular endothelial cell enlargement, and tubular damage with interstitial fibrosis.^[149]^ Various glomerular diseases following HSCT have been reported but are relatively uncommon. Kidney biopsy may be indicated and needed in some cancer cases, although there is much doubt about the benefit of the result. This topic needs further studies to assess the role of kidney biopsy benefits in malignancy-associated CKD.

The onset of renal failure often occurs several months after HSCT, which is characteristic of radiation-induced kidney injury. Compared to mucosal cells, kidney cells have a far slower rate of turnover, which causes radiation damage to manifest much later.^[150]^ Renal shielding during whole-body irradiation is moderately protective.^[151]^ Radiotherapy dose and concomitant cytotoxic treatment are considered significant in kidney damage.^[152]^ It has been thought that sirolimus may be linked to a greater incidence of TMA when combined with calcineurin inhibitor medication; however, this is often reversible.^[153]^ An angiotensin convertase enzyme genotype in HSCT-associated renal failure genes may affect renal damage; however, the findings were only marginally significant.^[152]^ Further studies to analyze the patient’s pre- and post-HSCT history carefully the underlying causes and prevention methods of CKD. It is crucial to analyze the patient’s pre- and post-HSCT history carefully. The following should be considered: kind of HSCT, type of conditioning treatment plan (specifically, whole-body irradiation usage and dosage), and level of nephrotoxin exposure (e.g., prolonged treatment with amphotericin).

For CKD patients, general therapy, including HTN management, should follow the recommendations. Patients with various kinds of CKD may frequently have anemia and hyperkalemia, necessitating more intensive treatment.^[154]^ Angiotensin-converting enzyme inhibitor or angiotensin receptor blockers, a low-potassium diet, diuretics, and low-dose sodium polystyrene, may be used.^[155]^ A small percentage of people may develop ESRD, and generally, these patients may do worse on hemodialysis than those with ESRD due to other causes.^[156]^ However, later reports showed successful kidney transplantation after bone marrow transplantation.^[157]^ Suitability for a kidney transplant must be determined case-by-case basis; it is important to note this because of the allograft’s immunologic tolerance. It is well-documented in nonrenal solid organ transplantation and autoimmune illness that prolonged use of these calcineurin inhibitors after HSCT most certainly causes CKD.^[158]^ Fortunately, most HSCT recipients stop taking calcineurin inhibitors after 4-12 months. In many HSCT patients, the impact of this medication class on CKD is likely to be minimal.

## 11. Prevention of cancer-induced nephropathy

A cancer patient with AKI should be assessed as any other patient with a hospital-acquired AKI. Further exposure to nephrotoxic medications should be avoided wherever possible. In the case of severe AKI after HSCT, no randomized controlled studies have compared continuous treatments with intermittent hemodialysis. Whatever the treatment method, it should be recognized that patients who have significant liver and renal failure have exceedingly poor prognoses. Continuous therapies have at least 2 potential benefits; there is some evidence that they are associated with increased intrarenal pressure in cardiac transplanted patients.^[159]^ Low platelet and neutropenia increase the risk of bleeding and infection, challenging vascular access. Hydration is the key to preventing and treating AKI in malignancy-induced nephropathy. Prevention of TLS, high serum uric acid, phosphate, and calcium is essential to diminish the risk of AKI. Early diagnosis of malignant diseases that directly affect the kidneys is necessary; however, the best method is a renal biopsy, but unfortunately is not usually possible due to bleeding risks or insufficient resources. Physicians should be aware of contrast-induced, direct radio, and chemotherapy-induced nephropathy. Adjusted doses of radiation and chemotherapy are essential to prevent CKD progression and AKI on top of CKD. Dialysis modalities are sometimes indicated. PET has been advocated in some cases of TMA, although there is conflicting data about its effectiveness.

## 12. Conclusions

AKI occurs uncommonly in cancer patients due to cancer or therapy. There are significant improvements in treating cancer patients, which have been shown to extend their lifespan. Rapid detection of kidney injury and proper management is essential to maintain the improvements in outcomes linked to chemotherapy and cancers.

AKI in cancer has multiple causes. Clinicians should be aware of all the potential causes of AKI, and when in doubt and the cause cannot be established, a kidney biopsy should be considered if there are no contraindications.

CKD is a known complication in cancer patients due to different causes, including medications; therefore, avoiding nephrotoxic agents and standardizing the doses of drugs is essential to minimize the risk of CKD.

Finally, given that AKI has the potential to impair outcomes considerably and invariably restrict the range of cancer medications’ accessibility, all attention should be paid to preventing AKI in the first place whenever feasible, as holding or avoiding anticancer therapy affects patient improvement and survival.

## Acknowledgments

We thank the Qatar National Library and Internal Medicine Residency Program of Hamad Medical Corporation for their scientific support.

## Author contributions

**Conceptualization:** Elmukhtar Habas, Raza Akbar, Amnna Rayani.

**Methodology:** Elmukhtar Habas, Kalifa Farfar, Nada Arrayes.

**Project administration:** Elmukhtar Habas, Aisha Aladab.

**Software:** Hafidh Ghazouani.

**Supervision:** Elmukhtar Habas, Amnna Rayani.

**Validation:** Elmukhtar Habas.

**Visualization:** Aml Habas, Gamal Alfitori, Eshrak Habas, Yaqeen Magassabi.

**Writing – original draft:** Elmukhtar Habas, Raza Akbar, Kalifa Farfar, Nada Arrayes, Aml Habas, Amnna Rayani, Gamal Alfitori, Eshrak Habas, Hafidh Ghazouani, Aisha Aladab.

**Writing – review & editing:** Elmukhtar Habas, Raza Akbar, Amnna Rayani, Abdel-Naser Elzouki.
